# An improved survivability prognosis of breast cancer by using sampling and feature selection technique to solve imbalanced patient classification data

**DOI:** 10.1186/1472-6947-13-124

**Published:** 2013-11-09

**Authors:** Kung-Jeng Wang, Bunjira Makond, Kung-Min Wang

**Affiliations:** 1 Department of Industrial Management, National Taiwan University of Science and Technology, Taipei 106, Taiwan; 2 Faculty of Commerce and Management, Prince of Songkla University, Trang, Thailand; 3 Department of Surgery, Shin-Kong Wu Ho-Su Memorial Hospital, Taipei, Taiwan

**Keywords:** Breast cancer, Decision tree, Logistic regression, Imbalanced data, Synthetic minority over-sampling, Cost-sensitive classifier technique

## Abstract

**Background:**

Breast cancer is one of the most critical cancers and is a major cause of cancer death among women. It is essential to know the survivability of the patients in order to ease the decision making process regarding medical treatment and financial preparation. Recently, the breast cancer data sets have been imbalanced (i.e., the number of survival patients outnumbers the number of non-survival patients) whereas the standard classifiers are not applicable for the imbalanced data sets. The methods to improve survivability prognosis of breast cancer need for study.

**Methods:**

Two well-known five-year prognosis models/classifiers [i.e., logistic regression (LR) and decision tree (DT)] are constructed by combining synthetic minority over-sampling technique (SMOTE) ,cost-sensitive classifier technique (CSC), under-sampling, bagging, and boosting. The feature selection method is used to select relevant variables, while the pruning technique is applied to obtain low information-burden models. These methods are applied on data obtained from the Surveillance, Epidemiology, and End Results database. The improvements of survivability prognosis of breast cancer are investigated based on the experimental results.

**Results:**

Experimental results confirm that the DT and LR models combined with SMOTE, CSC, and under-sampling generate higher predictive performance consecutively than the original ones. Most of the time, DT and LR models combined with SMOTE and CSC use less informative burden/features when a feature selection method and a pruning technique are applied.

**Conclusions:**

LR is found to have better statistical power than DT in predicting five-year survivability. CSC is superior to SMOTE, under-sampling, bagging, and boosting to improve the prognostic performance of DT and LR.

## Background

The need to monitor the survivability of breast cancer patients is threefold. First, breast cancer is one of the most critical cancers [1] and is a major cause of cancer death among women. DeSantis et al. [2] reported that in 2011, around 230,480 American women were diagnosed with invasive breast cancer and 39,520 breast cancer patients died. Second, the survivability of breast cancer patients has a significant impact on healthcare expenses and planning for both the government and private sectors. Third, the survivability of most common cancers (e.g., breast, prostate, lung, and colorectal) has changed over time, increasing continuously over the long term [3] because of the recent advances in cancer diagnosis and treatments, which reduce mortalities and increase survival time. Although many previous studies have been conducted, constant monitoring is still necessary. Thus, the survivability of breast cancer patients without bias is a critical task for the healthcare system.

Recently, artificial-intelligence-based data-mining techniques have been comprehensively used to predict the survivability of breast cancer patients. Lundin et al. [4] used the artificial neural network (ANN) to predict breast cancer survival in Turku, Finland, from 1945 to 1984. Soria et al. [5] compared three classifiers-naive Bayes algorithm, C4.5 DT, and multilayer perceptron function-to evaluate the most suitable technique for predicting the survivability of breast cancer patients from the Nottingham Tenovus Primary Breast Carcinoma Series. Khan et al. [6] used fuzzy DTs to predict breast cancer survivability. Chang and Liou [7] investigated the application of ANN, DT, logistic regression (LR), and genetic algorithm in the prognosis models of breast cancer acquired from patients at the University of Wisconsin.

Surveillance, Epidemiology, and End Results (SEER) data have been recognized and applied for breast cancer prognosis. Delen et al. [8] used the SEER database from 1973 to 2000, and studied breast cancer survivability using C5 decision tree, LR, and ANN. The five-year survival was 46% and their DT-based model was the best predictor with 93.62% accuracy. In comparison, the accuracy of ANN and LR were 91.21% and 89.20%, respectively. Bellaachia and Guven [9] used SEER data from 1973 to 2002 to predict breast cancer survivability and to compare naive Bayes network (BN), back-propagated ANN, and C4.5 DT; the real survivability was 76.80%. Their resulting decision trees (C4.5) had the best classification with 86.70% accuracy, followed by ANN and BN with 86.50% and 84.50% respectively. Endo et al. [10] used the SEER data set from 1992 to 1997. They proposed several models (i.e., LR, J48 DT, DT with naive BN, ANN, naive BN, BN, and ID3 DT) to predict the five-year survivability of breast cancer patients; the survivability was 81.50%. Their study showed that LR has the highest accuracy (85.80 ± 2%). Liu et al. [11] used DT-based predictive models for breast cancer survivability, concluding that the survival rate of patients was 86.52%. They employed the under-sampling technique and bagging algorithm to deal with the imbalanced problem, thus improving the predictive performance. These studies are comparatively summarized in Table 1.

**Table 1:** Breast cancer survival prognosis researches using SEER data

**Sources**	**Class distribution**	**Classifier methods**	**Accuracy performances**
Delen et al. [8]	Survival: 46%	C5 DT	93.62%
	Non-survival: 54%	ANN	91.21%
		LR	89.20%
Bellaachia and Guven [9]	Survival: 76.80%	C4.5 DT	86.70%
	Non-survival: 23.20%	ANN	86.50%
		Naïve BN	84.50%
Endo et al. [10]	Survival: 81.50%	LR	85.80%
	Non-survival: 18.50%	J48 DT	85.60%
		DT (with naïve Bayes)	84.20%
		ANN	84.50%
		Naïve BN	83.90%
		BN	83.90%
		ID3 DT	82.30%
Liu et al. [11]	Survival: 86.52%	C5 DT	88.05% (AUC = 0.607)
	Non-survival: 13.48%	Under-sampling + C5 DT	74.22% (AUC = 0.748)
		Bagging algorithm + C5 DT	76.59% (AUC = 0.768)

The studies using SEER data reveal two interesting points. First, the results proposed by previous studies that used DTs and LR to predict five-year survivability for breast cancer patients are controversial. Delen et al. [8] concluded that DT is more accurate than LR for breast cancer survivability, whereas Endo et al. [10] stated that the performance of LR is better than DT. Second, data mining methods were applied to a balanced data set in Delen et al. [8], whereas other studies, except Liu et al. [11], did not deal with imbalanced data which affected the performance of those methods. Owing to the conflicting results for predicting breast cancer survivability using LR and DT, and the imbalanced data situation, further investigation is required.

Several researchers argued that the imbalanced data problem will harm the performance of standard data mining methods [12-17]. Although researchers have devoted efforts to study imbalanced data sets, the subject remains unsolved: the number of survival and non-survival patients is obviously unequal, such as in the studies of Bellaachai and Guven [9], Endo et al. [10], and Khan et al. [6]. Liu et al. [11] employed the under-sampling technique and bagging algorithm to deal with imbalanced data and showed that the predictive performance is improved. However, under-sampling could lose information of the majority class, thereby reducing the predictive performance [18,19].

In recent years, a number of approaches are available to deal with imbalanced data problem. Re-sampling approaches which can be categorized into three groups: under-sampling method, over-sampling method, and hybrids method are useful approaches to balance the data set. Moreover, they are independent of the underlying classifier [20]. Random under-sampling and over-sampling are the simplest pre-processing approaches. Several empirical studies proved that random under-sampling is better than random over-sampling [21]. Synthetic minority over-sampling technique (SMOTE) proposed by Chawla et al. [22], is a well-known over-sampling method employed in data pre-processing, for example, by Zhao et al. [23], Pelayo and Dick [24], Kamei et al. [25], and Gu et al. [14]. Cost-sensitive learning (CSL) is a learning approach in data mining that considers the misclassification costs. The CSL minimizes the misclassification costs. Mostly, standard classification methods implicitly assume that all misclassification errors cost equally but it is not true in many applications. For example, in the medical problem, the classification of the presence of cancer in patients as the absence is more serious than the opposite misclassification because cancer patients will not be able to undergo appropriate treatments and will likely die [26]. Furthermore, bagging and boosting are ensemble learning methods and often adopt to the imbalanced data set problem. They improve the performance of single classifier by building several classifiers from the training data set and aggregating their predictions when unknown instances exist [20].

The present study predicts the five-year survivability of breast cancer patients by conducting a comparative study of DT and LR models. These models are constructed by combining SMOTE, cost-sensitive classifier technique (CSC), under-sampling, bagging, and boosting. Feature selection method is used to select relevant variables, and pruning technique is applied to obtain low information-burden models. Analysis of variance is used to detect the differences of these models and Tukey’s HSD test specifies which models are distinctive.

## Methods

### Data and pre-processing

This study uses data from the SEER_1973_2007_TEXTDATA [27] stored in four sub-directories, each of which consists of nine ASCII text files. The original data set has 973,125 records and 118 variables. The data of patients diagnosed from 1988 to 2002 are used to predict the five-year survivability for breast cancer patients because the follow-up cut-off date for this SEER data is December 31, 2007, and several variables (i.e., “Extent of Disease” and “AJCC stage of cancer” which are important to survivability prognosis modeling, as stated by Delen et al. [8]; Bellaachia & Guven [9], Khan et al. [6], and Liu et al. [11]) have only been recorded since 1988. Agrawal et al. [28] also selects the same period of data from the SEER database for lung cancer study.

Data pre-processing is crucial for data mining. It follows four principles: data cleaning, data integration, data transformation, and data reduction. The data are needed to resolve incompleteness and they undergo cleaning before application. In the current study, the following records are removed: (i) outliers, which are unusual data values that can seriously affect the models produced; for instance, the values of "Tumor size" greater than 200 mm are unusual because of obviously misrecorded data (also refer to Han & Kamber [29]); (ii) males, because this study focuses on breast cancer in females; and (iii) the instances that did not survive five years from the diagnosis date and have a recorded cause of death other than breast cancer. The remaining instances are indicated as survival if they survived five years after the diagnosis date; otherwise they are indicated as non-survival.

The resulting total number of records is 215,221, and the data set is pre-classified into two groups by their “survival” and “non-survival” attributes (Table 2). A binary target variable is defined as 1 (non-survival) and 0 (survival). Note that this is an imbalanced data set.

**Table 2:** Cancer survivability class distribution

**Class**	**Number of records**	**Percentage**
Survival (denote as 0)	195,172	90.68%
Non-survival (denote as 1)	20,049	9.32%
Total	215,221	100%

### Feature selection

According to our survey on features, as shown in Additional file 1: Table S1), the commonly selected (relevant) predictor variables for survivability in previous studies are summarized in Additional file 1: Table S2, and their descriptive statistics are computed. Using a large number of predictor variables cannot guarantee the performance of prognosis models if these variables are correlated with one another [30,31]. Applying variable selection approaches to the data set can improve the prognostic performance; provide faster and more cost-effective prognosis; and offer better understanding of the underlying process that generates the data [32]. In this study, we use a correlation-based feature subset selection method to select the predictor variables from the 20 variables identified in the literature. The predictor variables to have high correlation with the target variable but have low inter-correlation are selected. The correlation-based feature selection method [31] ranks feature subsets based on a heuristic evaluation function as follows: $M_{s}=\frac{k\bar{r_{\mathrm{cf}}}}{\sqrt{k+k\left(k-1\right)\bar{r_{\mathrm{ff}}}}}$, where *M*_*s*_ is the heuristic merit of the feature subset *S* containing *k* features, $\bar{r_{\mathrm{cf}}}$ is the average feature-class correlation, and $\bar{r_{\mathrm{ff}}}$ is the average feature-feature inter-correlation. To implement the correlation-based feature subset selection, we use best-first search for the space of variable subsets. The selected predictor variables are shown in Table 3.

**Table 3:** Resulting predictor variables selected in this study

**Categorical variables**		**Number of distinct values**			
**Variable ID in the study**	**Label**				
re_v4	Race		27		
re_v20	Grade		4		
re_v24	Extension of disease		32		
re_sss	Site-specific surgery code		9		
re_v26	Lymph node involvement		9		
re_v102	Stage of cancer		4		
re_v104	SEER modified AJCC stage 3rd		9		
**Numerical variables**		**Mean**	**S.D.**	**Min.**	**Max.**
v23	Tumor size	20.70	16.24	0	200
v27	Number of positive nodes	1.44	3.69	0	79

### Synthetic minority over-sampling technique

SMOTE operates in feature space rather than data space. Using this approach, the number of instances for the minority class in the original data set is increased by creating new synthetic instances, which results in broader decision regions for the minority class as compared to over-sampling with replacement. Consequently, the over-fitting problem in the learning algorithm can be avoided [14,15,22]. The new synthetic samples are created depending on the amount of over-sampling required (%) and the number of nearest neighbors (*k*). The procedures to create new synthetic instances for continuous features and nominal features are different.

The new synthetic samples for continuous features are generated through the following steps [12]:

Step 1: For each instance in the minority class, compute the distance between a feature vector of the instance and one of its *k* nearest neighbors.

Step 2: Multiply the distance obtained in Step 1 by a random number between 0 and 1.

Step 3: Add the value obtained from Step 2 to the original feature vector, which will yield a new synthetic instance given by

(1) $$\;x_{\mathrm{new}}=x+\delta \cdot \left(x_{i}‒x\right)$$

where *x*_*new*_ represents a new synthetic sample, *x* is denoted by a feature vector of each instance in the minority class, *x*_*i*_ is the i*th* selected nearest neighbor of *x*, and *δ* is a random number between 0 and 1. For example, given *β*% = 300% and *k* = 5, we have to generate three new synthetic instances for an original instance. The aforementioned three steps are repeated three times. Each time a new synthetic instance is created, one of the five nearest neighbors of *x* is randomly chosen.

For nominal features, synthetic instance generation is carried out through the following steps [12]:

Step 1: Obtain the majority vote between the features under consideration and its *k* nearest neighbors for the nominal feature value. In the case of a tie, choose at random.

Step 2: Assign the obtained value to the new synthetic minority class sample. For example, a set of features of a sample is {a, b, c, d, e} and the two nearest neighbors have the sets of features are {a, f, c, g, n} and {h, b, c, d, n}. Thus, the new synthetic sample has a set of the features, which is {a, b, c, d, n} [33].

### Cost-sensitive learning

CSL is an algorithm used to deal with the imbalanced data problem by considering misclassification costs. It can be categorized into two methods: direct and meta-learning [26]. The present study focuses on the wrapper method that converts any cost-insensitive algorithm into a cost-sensitive one without actually modifying the algorithm. The costs are not limited to finance, but also to time loss, the severity of an illness, and so on. The purpose of the learning is to build a model with minimum misclassification total costs.

For a binary class problem, the costs of classification can be given in a cost matrix (Table 4). False positive is misclassifying an actual negative instance as a positive; false negative is misclassifying an actual positive instance as a negative; and true positive and true negative are the correct classifications. The notation C(*i*, *j*) represents the cost of misclassifying an instance from actual class *i* as predicted class *j*. In addition, 1 represents positive class and 0 represents negative class [26].

**Table 4:** Cost matrix

		**Predicted class**	
		**Positive**	**Negative**
**Actual class**	**Positive**	C(1,1), or TP	C(1,0), or FN
	**Negative**	C(0,1), or FP	C(0,0), or TN

With the cost matrix, an instance is classified into the class that has the minimum expected cost. The expected cost *R*(*i*|*x*) of classifying an instance *x* into class *i* (by a classifier) can be expressed as

(2) $$R\left(i|x\right)=\;\sum_{j}P\left(j\left|x\right)\right)C\left(i,j\right)$$

where *P*(*j*|*x*) is the probability estimation of classifying an instance into class *j*.

We use CSC [34], a meta-learning method, because it is superior to MetaCost as shown in [35]. In addition, Afzal et al. [17] stated that MetaCost results in a large pre-processing time. CSC has two implementations [36]. The first is reweighting of training instances according to the total cost assigned to each class in the cost matrix, and the second is predicting the class with the minimum expected misclassification cost by using the values in the cost matrix [37]. The former is implemented for this study.

### Logistic regression

Logistic regression [38] is a statistical method used to describe the relation between predictor variables denoted by  ***x***^'^ = (*x*_1_, *x*_2_, …, *x*_*p*_) and a response variable, which is a categorical variable with two values (here, “survival” or “non-survival”).

The conditional probability of non-survival patient can be written as *P*(*Y* = 1|*x*) = *π*(*x*). Thus, the LR model for *p* predictor variables can be written as

(3) $$\pi \left(x\right)=\frac{e^{\left(\beta_{0}+\beta_{1}x_{1}+\beta_{2}x_{2}+\ldots +\beta_{p}x_{p}\right)}}{1+e^{\left(\beta_{0}+\beta_{1}x_{1}+\beta_{2}x_{2}+\ldots +\beta_{p}x_{p}\right)}}$$

where 0 ≤ *π*(*x*) ≤ 1

A useful transformation of LR is the logit transformation, defined as

(4) $$\;g\left(x\right)=\ln\left(\frac{\pi \left(x\right)}{1-\pi \left(x\right)}\right)=\beta_{0}+\beta_{1}x_{1}+\beta_{2}x_{2}+\ldots +\beta_{p}x_{p}$$

The parameters ***β*** = *β*_0_,  *β*_1_, *β*_2_, …, *β*_*p*_ are obtained by maximum likelihood method. This method finds the estimators of parameters that maximize the likelihood function:

(5) $$l\left(\beta \right)=\prod_{i=1}^{n}\pi {\left(x_{i}\right)}^{y_{i}}{\left[1-\pi \left(x_{i}\right)\right]}^{1-y_{i}}$$

The log likelihood of (5) is defined as

(6) $$L\left(\beta \right)=\ln l\left(\beta \right)=\sum_{i=1}^{n}\left\{y_{i}\ln\left[\pi \left(x_{i}\right)\right]+\left(1-y_{i}\right)\left[1-\pi \left(x_{i}\right)\right]\right\}$$

To find the maximum likelihood estimators, *L*(*β*) is differentiated with respect to each parameter, and then the resulting terms are set as equal to zero. Other methods such as Newton's method can be utilized.

The odds ratio (OR) is widely used to interpret the model. It associates with one unit change in *x*_*j*_ represented with $\;e^{\left(\beta_{j}\right)}$.

The highly correlated variable or multicollinearity problem leads to unstable parameter estimation. Ridge regression method can decrease the impact of multicollinearity in ordinary least squares regression, and is applied to logistic regression to find the ridge estimator. Ridge estimator *β*_*r*_ is defined as [39]

(7) $$\beta_{r}={\left(X^{'}\mathrm{VX}+kI\right)}^{-1}X'\mathrm{VX}\beta_{\mathrm{mle}}$$

where *β*_*mle*_ is the maximum likelihood estimator of *β*, *V* is the diagonal matrix of the maximum likelihood estimators of success probabilities, *I* is the identity matrix, and *k* is the ridge constant.

### Decision tree

This study also adopts the J48 DT algorithm to predict the survivability of breast cancer patients. J48 is a release of C4.5, which has high accuracy, comprehensibility, and stability. In addition, C4.5, which was developed from the ID3 algorithm, deals with problems of missing data, continuous data, pruning rules, and splitting criterion [40]. Throughout the current paper, we use the DT notation instead of J48 DT.

### Model evaluation

To evaluate the performance of the proposed prognosis models, a full data set is divided into two sets: training and testing sets. In this study, a 10-fold cross-validation is employed so that the bias caused by random sampling for training and testing sets can be reduced [34]. In this way, a full data set is divided into 10 independent folds (subsets); each fold is approximately one-tenth of the full data set (with approximately one-tenth of survival and one-tenth of non-survival). Nine of the ten subsets are combined and used as the training set, and the remaining subset is used as the testing set. Each of the 10 subsets is used once as the testing set to evaluate the performance of the model, which is built from the combination of the other remaining subsets.

The sensitivity, specificity, accuracy, area under the receiver operating characteristic curve (AUC), and g-mean are used to evaluate the prognosis performance of the models.

These measures are as follow:

(8) $$\mathrm{Sensitivity}=\frac{\mathrm{TP}}{\mathrm{TP}+\mathrm{FN}}$$

(9) $$\mathrm{Specificity}=\frac{\mathrm{TN}}{\mathrm{TN}+\mathrm{FP}}$$

(10) $$\mathrm{Accuracy}=\frac{\mathrm{TN}+\mathrm{TP}}{\mathrm{TN}+\mathrm{FP}+\mathrm{TP}+\mathrm{FN}}$$

(11) $$g‒\mathrm{mean}=\sqrt{\mathrm{sensitivity}\times \mathrm{specificity}}$$

(12) $$\mathrm{AUC}=\frac{1+TP_{\mathrm{rate}}-FP_{\mathrm{rate}}}{2}$$

where *TP* denotes the true positives, *TN* denotes the true negatives, *FP* denotes the false positives, and *FN* denotes the false negatives. These values are often displayed in a confusion matrix, as presented in Table 5.

**Table 5:** Confusion matrix

		**Predicted class**	
		**Non-survival**	**Survival**
**Actual class**	**Non-survival**	TP	FN
	**Survival**	FP	TN

### Experiment framework

**Figure 1:**
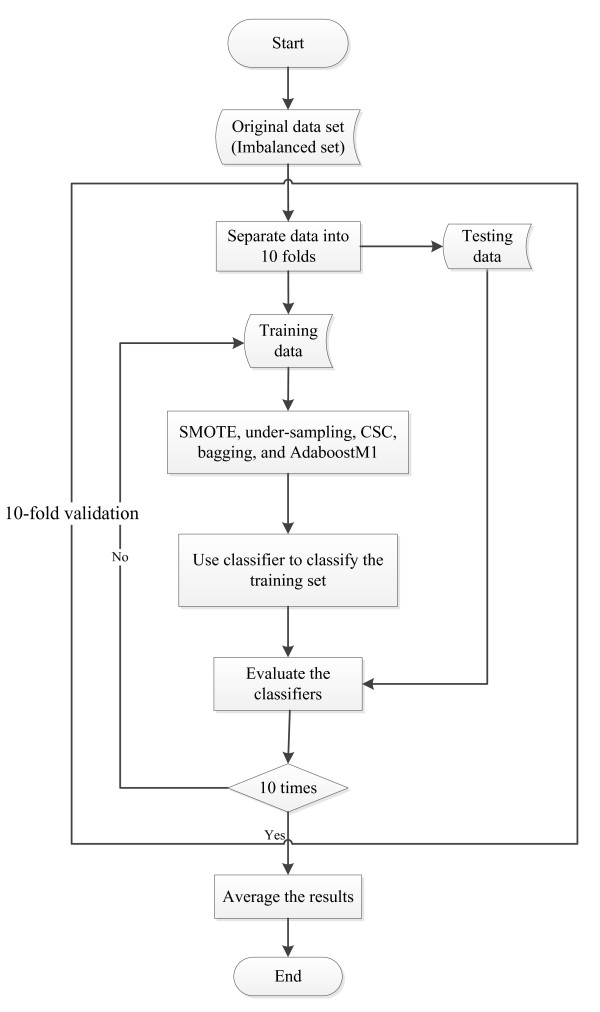
Flow diagram of SMOTE, CSC, under-sampling, bagging, and boosting implementation.

The data set after data pre-processing is imbalanced; survival patients outnumber the non-survival patients. When standard data mining methods are implemented to the imbalanced data set, they will be overwhelmed by the instances in the majority class, and the instances in the minority class will be ignored, resulting in high accuracy for the majority class but poor accuracy for the minority class consequently [41]. SMOTE and CSC approaches are utilized to solve this problem. Moreover, some techniques widely used in imbalanced domain such as under-sampling, bagging, and boosting are also used in this study. The implementations of SMOTE, CSC, under-sampling, bagging, and boosting are shown in Figure 1.

SMOTE is implemented to the training data set to increase the number of instances in the minority class by creating new synthetic instances. The number of new synthetic instances increases depending on the amount of over-sampling required. For this study, the amount of over-sampling is set at 900% because the numbers of instances in majority and minority classes will be approximately balanced. Moreover, because of the wide use of five nearest neighbors in various studies, we use five nearest neighbors in this study as well.

CSC is implemented to the training data set so that each training instance is reweighted according to the costs assigned to each class. We identify the misclassification cost of a model by following Lopez et al. [42]; that is, the cost of misclassifying a non-survival patient as survival equals the imbalance ratio, which is the ratio of the number of instances of the majority class and the minority class. Therefore, the cost of misclassifying a non-survival patient as a survival patient is 10 [i.e., C(1,0) = 10], whereas the cost of misclassifying a survival patient as a non-survival patient is 1 [C(0,1) = 1]. The cost of correct classification in each class is 0 [C(1,1) = C(0,0) = 0].

Under-sampling, bagging, and boosting are also used for comparison. Under-sampling is implemented to the training data set to balance class distribution through the random elimination of majority class instances. In this experiment, we define the class distribution is uniform distribution. Bagging and boosting are ensemble methods. Bagging sample subsets from the training set to form different classifiers and aggregate their predictions to make final prediction. Boosting uses all data set to train each classifier serially. After each round, it gives more focus to difficult instances and assigns the weight to each individual classifier depending on its overall accuracy. Finally, each classifier gives a weight vote to the new instance and the class label is selected by majority. The implementation of both bagging and boosting follows the default setting. AdaboostM1 is used for boosting method. These methods are implemented on Weka.

All models consisting of the combination of SMOTE, under-sampling, bagging, AdaboostM1 with DT and LR models, and of CSC wrapping with DT and LR models are implemented. These models in form of acronyms along with the descriptions are presented in Table 6. Tenfold cross-validation is applied to evaluate the performances of the models. The sensitivity, specificity, g-mean, and AUC are calculated as the average of the 10 individual indexes.

**Table 6:** All studied models in form of acronyms along with the descriptions

**Acronym**	**Description**
DT_9	Decision tree algorithm with 9 predictor variables
LR_9	Logistic regression algorithm with 9 predictor variables
S_DT_9	Decision tree algorithm with 9 predictor variables, pre-processed by using the SMOTE
S_LR_9	Logistic regression algorithm with 9 predictor variables, pre-processed by using the SMOTE
S_DT_10	Decision tree algorithm with 10 predictor variables proposed by Endo et al. [10], pre-processed by using the SMOTE
S_LR_10	Logistic regression algorithm with 10 predictor variables proposed by Endo et al. [10], pre-processed by using the SMOTE
S_DT_16	Decision tree algorithm with 16 predictor variables proposed by Delen et al. [8], pre-processed by using the SMOTE
S_LR_16	Logistic regression algorithm with 16 predictor variables proposed by Delen et al. [8], pre-processed by using the SMOTE
S_DT_20	Decision tree algorithm with 20 predictor variables, pre-processed by using the SMOTE
S_LR_20	Logistic regression algorithm with 20 predictor variables, pre-processed by using the SMOTE
S_pDT	Pruning decision tree algorithm pre-processed by using the SMOTE
S_rLR	Logistic regression algorithm pre-processed by using the SMOTE (This model is constructed by the same predictor variables as in S_pDT)
C_DT_9	Decision tree algorithm with 9 predictor variables, wrapped with CSC
C_LR_9	Logistic regression algorithm with 9 predictor variables, wrapped with CSC
C_DT_10	Decision tree algorithm with 10 predictor variables proposed by Endo et al. [10], wrapped with CSC
C_LR_10	Logistic regression algorithm with 10 predictor variables proposed by Endo et al. [10], wrapped with CSC
C_DT_16	Decision tree algorithm with 16 predictor variables proposed by Delen et al. [8], wrapped with CSC
C_LR_16	Logistic regression algorithm with 16 predictor variables proposed by Delen et al. [8], wrapped with CSC
C_DT_20	Decision tree algorithm with 20 predictor variables, wrapped with CSC
C_LR_20	Logistic regression algorithm with 20 predictor variables, wrapped with CSC
C_pDT	Pruning decision tree algorithm wrapped with CSC
C_rLR	Logistic regression algorithm wrapped with CSC (This model is constructed by the same predictor variables as in C_pDT)
U_DT_9	Decision tree algorithm with 9 predictor variables, pre-processed by using the under-sampling approach
U_LR_9	Logistic regression algorithm with 9 predictor variables, pre-processed by using the under-sampling approach
U_DT_10	Decision tree algorithm with 10 predictor variables proposed by Endo et al. [10], pre-processed by using the under-sampling approach
U_LR_10	Logistic regression algorithm with 10 predictor variables proposed by Endo et al. [10], pre-processed by using the under-sampling approach
U_DT_16	Decision tree algorithm with 16 predictor variables proposed by Delen et al. [8], pre-processed by using the under-sampling approach
U_LR_16	Logistic regression algorithm with 16 predictor variables proposed by Delen et al. [8], pre-processed by using the under-sampling approach
U_DT_20	Decision tree algorithm with 20 predictor variables, pre-processed by using the under-sampling approach
U_LR_20	Logistic regression algorithm with 20 predictor variables, pre-processed by using the under-sampling approach
U_pDT	Pruning decision tree algorithm pre-processed by using the under-sampling approach
U_rLR	Logistic regression algorithm pre-processed by using the under-sampling approach (This model is constructed by the same predictor variables as in U_pDT)
Ba_DT_9	Decision tree algorithm with 9 predictor variables, combined with bagging
Ba_LR_9	Logistic regression algorithm with 9 predictor variables, combined with bagging
Ba_DT_10	Decision tree algorithm with 10 predictor variables proposed by Endo et al. [10], combined with bagging
Ba_LR_10	Logistic regression algorithm with 10 predictor variables proposed by Endo et al. [10], combined with bagging
Ba_DT_16	Decision tree algorithm with 16 predictor variables proposed by Delen et al. [8], combined with bagging
Ba_LR_16	Logistic regression algorithm with 16 predictor variables proposed by Delen et al. [8], combined with bagging
Ba_DT_20	Decision tree algorithm with 20 predictor variables, combined with bagging
Ba_LR_20	Logistic regression algorithm with 20 predictor variables, combined with bagging
Ba_pDT	Pruning decision tree algorithm combined with bagging
Ba _rLR	Logistic regression algorithm combined with bagging (This model is constructed by the same predictor variables as in Ba_pDT)
Ad_DT_9	Decision tree algorithm with 9 predictor variables, combined with AdaboostM1
Ad_LR_9	Logistic regression algorithm with 9 predictor variables, combined with AdaboostM1
Ad_DT_10	Decision tree algorithm with 10 predictor variables proposed by Endo et al. [10], combined with AdaboostM1
Ad_LR_10	Logistic regression algorithm with 10 predictor variables proposed by Endo et al. [10], combined with AdaboostM1
Ad_DT_16	Decision tree algorithm with 16 predictor variables proposed by Delen et al. [8], combined with AdaboostM1
Ad_LR_16	Logistic regression algorithm with 16 predictor variables proposed by Delen et al. [8], combined with AdaboostM1
Ad_DT_20	Decision tree algorithm with 20 predictor variables, combined with AdaboostM1
Ad_LR_20	Logistic regression algorithm with 20 predictor variables, combined with AdaboostM1
Ad_pDT	Pruning decision tree algorithm combined with AdaboostM1
Ad_rLR	Logistic regression algorithm combined with AdaboostM1 (This model is constructed by the same predictor variables as in Ad_pDT)

Statistical analysis is utilized to find the differences in predictive performance among the models. The differences in the performances of the models are detected by using ANOVA test, in which the model is treated as a factor. Multiple comparison tests are also conducted using Tukey’s HSD test to identify the distinctive models. The significant level for the entire differences test is defined at 0.05.

## Results

### Efficiency of all techniques

To show that SMOTE, CSC, under-sampling, bagging and AdaboostM1 can improve the predictive performance of the original models, we input nine prognosis variables (see Table 3) for S_DT_9, S_LR_9, C_DT_9, C_LR_9, U_DT_9, U_LR_9, Ba_DT_9, Ba_LR_9, Ad_DT_9, and Ad_LR_9 model constructions. Then the results are compared with the standard data mining models (i.e., DT_9 and LR_9). The comparative results are shown in Table 7. We find that although the specificity decreases slightly (loss majority prognosis accuracy) when applying SMOTE, CSC, and under-sampling, the sensitivity and g-mean are improved; while AUC values indicate that the performance of DT and LR when applying SMOTE and AdaboostM1 are slightly decreased. The highest g-mean value corresponds to C_LR_9 model.

**Table 7:** The comparative results of models using all techniques and standard data mining models

**Model**	**Accuracy**	**Sensitivity**	**Specificity**	**g-mean**	**AUC**
DT_9	0.912	0.140	0.991	0.374	0.772
LR_9	0.913	0.156	0.990	0.394	0.829
S_DT_9	0.791	0.475	0.823	0.626	0.700
S_LR_9	0.759	0.645	0.771	0.705	0.783
C_DT_9	0.772	0.669	0.792	0.727	0.758
C_LR_9	0.752	0.752	0.752	0.752	0.829
U_DT_9	0.748	0.748	0.749	0.748	0.798
U_LR_9	0.749	0.732	0.767	0.749	0.825
Ba_DT_9	0.911	0.151	0.990	0.386	0.797
Ba_LR_9	0.913	0.157	0.990	0.394	0.829
Ad_DT_9	0.902	0.197	0.974	0.438	0.752
Ad_LR_9	0.913	0.157	0.990	0.394	0.787

ANOVA results (in Table 8) detect a significant difference among these models because the returned *p*-value (0.000) is lower than the defined *α*-value (0.05). The differences among models (in Table 9) are identified by Tukey’s HSD test, which lists the different models in different columns while the indifferent models are listed in the same column.

**Table 8:** ANOVA for average g-mean

**Sources of variance**	**Sum of squares**	**d.f.**	**Mean square**	**F**	***p*-value**
Between Groups	3.239	11	0.294	4335.466	0.000
Within Groups	0.007	108	0.000		
Total	3.246	119			

**Table 9:** Tukey’s HSD test for g-mean

**Model**	**Different subset**						
	**1**	**2**	**3**	**4**	**5**	**6**	**7**
DT_9	0.374						
Ba_DT_9	0.386	0.386					
LR_9		0.394					
Ba_LR_9		0.394					
Ad_LR_9		0.394					
Ad_DT_9			0.438				
S_DT_9				0.626			
S_LR_9					0.705		
C_DT_9						0.727	
U_DT_9							0.748
U_LR_9							0.749
C_LR_9							0.752

In Table 9, the results present that SMOTE, CSC, and under-sampling can significantly improve the predictive performance of both DT and LR; AdaboostM1 can only improve the predictive performance of DT; and bagging can improve the predictive performance of neither DT model nor LR model. However, the results show that LR outperforms DT in terms of g-mean value for all cases with and without the use of SMOTE, CSC, under-sampling, bagging and AdaboostM1. The C_LR_9 model has the highest g-mean.

### Efficiency of feature selection

Table 10 shows the comparative results of the DT and LR models constructed from four sets of prognosis variables: the nine prognosis variables (in Table 3), the variables previously used by Delen et al. [8], the variables previously used by Endo et al. [10], and all selected predictor variables (Additional file 1: Table S2). The variables previously used in Delen et al. [8] and Endo et al. [10] are chosen for this comparison because their studies proposed DT and LR models to predict five-year survivability for breast cancer using the SEER database as well. SMOTE, CSC, under-sampling, bagging, and AdaboostM1 are implemented to deal with the imbalanced data problem so that the comparative results are only influenced by the different sets of variables.

**Table 10:** The comparative results of models with feature selection using all techniques

**Model**	**Accuracy**	**Sensitivity**	**Specificity**	**g-mean**	**AUC**
S_DT_9	0.791	0.475	0.823	0.626	0.700
S_LR_9	0.759	0.645	0.771	0.705	0.783
S_DT_10	0.835	0.363	0.884	0.566	0.726
S_LR_10	0.772	0.492	0.800	0.627	0.720
S_DT_16	0.869	0.310	0.926	0.536	0.731
S_LR_16	0.796	0.471	0.830	0.623	0.727
S_DT_20	0.871	0.311	0.929	0.537	0.733
S_LR_20	0.791	0.476	0.824	0.626	0.726
C_DT_9	0.772	0.669	0.792	0.727	0.758
C_LR_9	0.752	0.752	0.752	0.752	0.829
C_DT_10	0.723	0.734	0.722	0.728	0.774
C_LR_10	0.723	0.766	0.719	0.742	0.818
C_DT_16	0.804	0.557	0.829	0.679	0.673
C_LR_16	0.662	0.810	0.647	0.724	0.814
C_DT_20	0.805	0.552	0.831	0.677	0.672
C_LR_20	0.591	0.824	0.567	0.684	0.787
U_DT_9	0.748	0.748	0.749	0.748	0.798
U_LR_9	0.749	0.732	0.767	0.749	0.825
U_DT_10	0.744	0.767	0.720	0.743	0.795
U_LR_10	0.743	0.762	0.724	0.743	0.817
U_DT_16	0.746	0.745	0.748	0.746	0.786
U_LR_16	0.749	0.727	0.771	0.749	0.826
U_DT_20	0.746	0.744	0.748	0.746	0.785
U_LR_20	0.753	0.743	0.764	0.753	0.829
Ba_DT_9	0.911	0.151	0.990	0.386	0.797
Ba_LR_9	0.913	0.157	0.990	0.394	0.829
Ba_DT_10	0.911	0.117	0.992	0.341	0.784
Ba_LR_10	0.911	0.126	0.991	0.354	0.818
Ba_DT_16	0.912	0.186	0.987	0.429	0.801
Ba_LR_16	0.913	0.177	0.989	0.418	0.829
Ba_DT_20	0.912	0.189	0.987	0.432	0.801
Ba_LR_20	0.914	0.187	0.989	0.430	0.835
Ad_DT_9	0.902	0.197	0.974	0.438	0.752
Ad_LR_9	0.913	0.157	0.990	0.394	0.787
Ad_DT_10	0.905	0.146	0.983	0.379	0.773
Ad_LR_10	0.911	0.112	0.993	0.334	0.783
Ad_DT_16	0.890	0.247	0.956	0.486	0.749
Ad_LR_16	0.914	0.177	0.989	0.418	0.779
Ad_DT_20	0.891	0.247	0.958	0.487	0.748
Ad_LR_20	0.914	0.180	0.990	0.422	0.794

ANOVA results in Table 11 detect the significant differences among the models because the returned *p*-value (0.000) is lower than the defined *α*-value (0.05). The result of Tukey’s HSD test in Table 12 shows that the performance of C_LR_9 constructed by using the nine prognosis variables is indifferent from U_LR_20 model constructed by using more variables (20 variables).

**Table 11:** ANOVA for average g-mean of models using feature selection

**Sources of variance**	**Sum of squares**	**d.f.**	**Mean square**	**F**	***p*-value**
Between Groups	8.994	39	0.231	2266.112	0.000
Within Groups	0.037	360	0.000		
Total	9.031	399			

**Table 12:** Tukey’s HSD test for g-mean of models using feature selection

**Model**	**Different subset**													
	**1**	**2**	**3**	**4**	**5**	**6**	**7**	**8**	**9**	**10**	**11**	**12**	**13**	**14**
Ad_LR_10	0.334													
Ba_DT_10	0.341	0.341												
Ba_LR_10		0.354												
Ad_DT_10			0.379											
Ba_DT_9			0.386											
Ba_LR_9			0.394											
Ad_LR_9			0.394											
Ad_LR_16				0.418										
Ba_LR_16				0.418										
Ad_LR_20				0.422	0.422									
Ba_DT_16				0.429	0.429									
Ba_LR_20				0.430	0.430									
Ba_DT_20				0.432	0.432									
Ad_DT_9					0.438									
Ad_DT_16						0.486								
Ad_DT_20						0.487								
S_DT_20							0.537							
S_DT_16							0.536							
S_DT_10								0.566						
S_LR_16									0.623					
S_DT_9									0.626					
S_LR_20									0.626					
S_LR_10									0.627					
C_DT_20										0.677				
C_DT_16										0.679				
C_LR_20										0.684				
S_LR_9											0.705			
C_LR_16												0.724		
C_DT_9												0.727	0.727	
C_DT_10												0.728	0.728	
C_LR_10													0.742	0.742
U_LR_10													0.743	0.743
U_DT_10													0.743	0.743
U_DT_16														0.746
U_DT_20														0.746
U_DT_9														0.748
U_LR_9														0.749
U_LR_16														0.749
C_LR_9														0.752
U_LR_20														0.753

The correlation-based feature subset selection method can reduce the information burden (i.e., the number of predictor variables), but still retain the quality of classification. This occurrence is in accordance with the study of Hall and Smith [30], which states that using a larger number of predictor variables cannot increase the performance of models for machine learning if these variables are correlated with one another.

### Feature pruning effect

To derive the fit model for five-year survivability, we use feature pruning by setting a confidence factor of 0.05. Each minimum number of instances at 15,000, 5000, 1,000, 1,000, and 15,000 is used to form the S_pDT, C_pDT, U_pDT, Ba_pDT, and Ad_pDT model, respectively. Then, S_rLR, C_ rLR, U_ rLR, Ba_ rLR, and Ad_ rLR models are constructed by the same predictor variables as in S_pDT, C_pDT, U_pDT, Ba_pDT, and Ad_pDT model in pair, respectively. The variables to form these models are selected by information entropy to predict five-year survivability. The comparative results are shown in Table 13.

**Table 13:** The comparative results of models using feature pruning

**Model**	**Accuracy**	**Sensitivity**	**Specificity**	**g-mean**	**AUC**
S_DT_9	0.791	0.475	0.823	0.626	0.700
S_LR_9	0.759	0.645	0.771	0.705	0.783
S_pDT	0.728	0.703	0.731	0.717	0.770
S_rLR	0.747	0.717	0.750	0.734	0.811
C_DT_9	0.772	0.669	0.792	0.727	0.758
C_LR_9	0.752	0.752	0.752	0.752	0.829
C_pDT	0.740	0.748	0.740	0.744	0.795
C_rLR	0.770	0.719	0.776	0.747	0.824
U_DT_9	0.748	0.748	0.749	0.748	0.798
U_LR_9	0.749	0.732	0.767	0.749	0.825
U_pDT	0.740	0.749	0.731	0.740	0.791
U_rLR	0.745	0.703	0.787	0.743	0.823
Ba_DT_9	0.911	0.151	0.990	0.386	0.797
Ba_LR_9	0.913	0.157	0.990	0.394	0.829
Ba_pDT	0.911	0.107	0.994	0.324	0.724
Ba_rLR	0.912	0.142	0.991	0.377	0.823
Ad_DT_9	0.902	0.197	0.974	0.438	0.752
Ad_LR_9	0.913	0.157	0.990	0.394	0.787
Ad_pDT	0.911	0.161	0.988	0.397	0.822
Ad_rLR	0.910	0.130	0.990	0.359	0.745

ANOVA test result (in Table 14) shows that the differences among these models exist because the *p*-value (0.000) is lower than the defined *α*-value (0.05).

**Table 14:** ANOVA for average g-mean value of models using feature pruning

**Sources of variance**	**Sum of squares**	**d.f.**	**Mean square**	**F**	***p*-value**
Between Groups	5.896	19	0.310	2062	0.000
Within Groups	0.027	180	0.000		
Total	5.923	199			

Tukey’s HSD test (in Table 15) shows some findings which are statistically significant: (i) when the CSC or SMOTE combined with feature selection and pruning process are employed, LR models outperform DT models in most cases. Likewise, LR and DT models perform equally when the under-sampling, feature selection, and pruning process are employed; (ii) the performance of DT and LR models that use CSC, SMOTE, and under-sampling combining with feature selection and pruning process are improved in terms of g-mean, but their performances are worse when bagging and AdaboostM1 combining with feature selection and pruning process are employed; (iii) the g-mean values show that C_rLR, U_DT_9, U_DT_9, and C_LR_9 models which have the indifferent performance are definitely different from the other models; it is worthwhile to note that C_LR_9 has the highest g-mean value among the four models.

**Table 15:** Tukey’s HSD test for g-mean of models using feature pruning

**Model**	**Different subset**								
	**1**	**2**	**3**	**4**	**5**	**6**	**7**	**8**	**9**
Ba_pDT	0.324								
Ad_rLR		0.359							
Ba_rLR		0.377	0.377						
Ba_DT_9			0.386						
Ba_LR_9			0.394						
Ad_LR_9			0.394						
Ad_pDT			0.397						
Ad_DT_9				0.438					
S_DT_9					0.626				
S_LR_9						0.705			
S_pDT						0.717	0.717		
C_DT_9							0.727	0.727	
S_rLR							0.734	0.734	0.734
U_pDT								0.740	0.740
U_rLR								0.743	0.743
C_pDT								0.744	0.744
C_rLR									0.747
U_DT_9									0.748
U_LR_9									0.749
C_LR_9									0.752

Some interesting points are observed between C_rLR and C_LR_9. First, C_rLR models need lesser information than C_LR_9 (four variables versus nine variables) on model construction. Second, C_rLR predicts five-year survivability with the lowest total number of misclassified instances (i.e., 49,446 instances) while the number of misclassified instances of C_LR_9 model is 53,340. Third, the C_rLR model has the highest accuracy in predicting the five-year survivability of breast cancer patients correctly.

### The improvement of the proposed method

In this study, the best method we proposed is the combination of CSC, a feature selection method, and a pruning process. To show the comparable results of our proposed method and the previous methods, we prepare the data sets from the same database and period as used by Delen et al. [8] and Endo et al. [10]. The proposed method and the previous methods are applied on those data sets. The results in Tables 16 and 17 show that our proposed method outperforms the previous methods in terms of g-mean and sensitivity.

**Table 16:** **The comparative results (data from the same database and period as used by Delen et al.**[8]**)**

**Model**	**Accuracy**	**Sensitivity**	**Specificity**	**g-mean**	**AUC**
Proposed method (C_rLR)	0.751	0.762	0.750	0.756	0.842
Previous method (LR)	0.903	0.272	0.985	0.517	0.849
Proposed method (C_pDT)	0.758	0.756	0.758	0.757	0.820
Previous method (DT)	0.903	0.279	0.984	0.524	0.769

**Table 17:** **The comparative results (data from the same database and period as used by Endo et al.**[10]**)**

**Model**	**Accuracy**	**Sensitivity**	**Specificity**	**g-mean**	**AUC**
Proposed method (C_rLR)	0.723	0.748	0.719	0.733	0.814
Previous method (LR)	0.897	0.226	0.988	0.472	0.832
Proposed method (C_pDT)	0.747	0.756	0.746	0.752	0.812
Previous method (DT)	0.896	0.214	0.988	0.460	0.793

## Discussion

The results demonstrated that CSC technique and sampling techniques (i.e., SMOTE and under-sampling) can significantly improve the performance of five-year prognosis models/classifiers for breast cancer patients (i.e., LR and DT). CSC technique often outperforms sampling techniques, this result is in accordance with the conclusion of McCarthy et al. [43]. While, the results show that AdaboostM1 can only improve the predictive performance of DT, and bagging cannot improve the predictive performance of DT and LR models.

This study proposes the best method to deal with imbalanced data set problem and to improve survivability prediction of breast cancer patients. The best method is the combination of CSC, a feature selection method, and a pruning process. CSC which solves the imbalanced data set problem by considering misclassification costs does not change the original data set. Moreover, feature selection method can improve the predictive performance of models by selecting the predictor variables most related to target variable; in addition, feature selection can solve the problem when there are correlated variables in the data set which harm the performance of the models.

## Conclusions

This study has employed more comprehensive and most current data than the previous studies on the prognosis of breast cancer patients. We obtain the following conclusions: (i) the proposed technique to solve imbalanced problem, feature selection and pruning process can significantly improve the performance of two well-known five-year prognosis models/classifiers for breast cancer patients (i.e., LR and DT); (ii) CSC is superior to the other methods in improving the prognosis performance of both DT and LR with an imbalanced data set; (iii) the correlation-based feature subset selection method and the feature pruning process using information entropy can reduce the informative burden (i.e., the number of predictor variables) but still retain the quality of classification; (iv) by considering the results (Tables 9, 12 and 15), the performance of LR models outperform the DT models when these models are solely implemented, and when they employ CSC and SMOTE techniques, feature selection, and/or pruning process; (v) bagging cannot improve the predictive performance of DT model and LR model; while AdaboostM1 can only improve the predictive performance of DT. However, the improvement of DT is lower than of DT wrapped with CSC; (vi) although under-sampling technique can deal with imbalanced data set, it is not as good as CSC in terms of g-mean. Moreover it can discard potentially useful data.

For the low information-burden models, our study shows that the C_LR_9 model has the highest g-mean, but the C_LR_9 and C_rLR models are equally powerful statistically. Interesting phenomena are observed: (i) the performances of C_rLR are similar to the C_LR_9 model, but the earlier need fewer variables; (ii) C_rLR gives the highest accuracy to predict the survivability of patients and has the lowest total number of misclassified instances.

Future research can investigate the embedding of SMOTE and CSC into alternative classifiers, such as advanced population-based algorithms, to improve the prediction performance of five-year survivability of breast cancer patients.

## Competing interests

The authors declare that they have no competing interests.

## Authors' contributions

KJW is the main author of this paper. He designed the experiments, supervised and revised the manuscript. BM prepared the data set, carried out the experiments, and drafted the manuscript. KMW provided continuous feedback on the paper. All authors read and approved the manuscript.

## Pre-publication history

The pre-publication history for this paper can be accessed here:

http://www.biomedcentral.com/1472-6947/13/124/prepub

## Supplementary Material

Additional file 1 **Appendix.** This file contains two tables. **Table S1**. shows the predictor variables for survivability in previous studies [6,8-11,44,45]. **Table S2**. shows the summary of predictor variables for survivability in the literature. Click here for file
