# Supplementary material for: An improved survivability prognosis of breast cancer by using sampling and feature selection technique to solve imbalanced patient classification data
Source: BMC Med Inform Decis Mak. 2013 Nov 9;13:124. doi: 10.1186/1472-6947-13-124 (PMC3829096; doi:10.1186/1472-6947-13-124)
Supplement: Additional file 1 — Appendix. This file contains two tables. Table S1. shows the predictor variables for survivability in previous studies [6,8-11,44,45]. Table S2. shows the summary of predictor variables for survivability in the literature. [file 1472-6947-13-124-S1.pdf]

Additional file 1: Appendix

Predictor variables for survivability

Table S1 Predictor variables for survivability in previous studies

Authors	Predictor variables	
	**Categorical variables**	**Continuous variables**
Delen et al. [8]	Race, Marital status, Primary site code, Histology, Behavior, Grade, Extension of disease, Lymph node involvement, Radiation, Stage of cancer, Site-specific surgery code	Age, Tumor size, Number of positive nodes, Number of Nodes, Number of primaries
Bellaachia and Guven [9]	Race, Marital status, Primary site code, Histologic type, Behavior code, Grade, Extension of tumor, Lymph node involvement, Radiation, Stage of cancer, Site-specific surgery code	Age, Tumor size, Number of positive nodes, Number of Nodes, Number of primaries
Endo et al. [10]	Race, Marital status, Behavior Code ICD-O-3, Grade, Radiation, SEER modified AJCC stage 3rded, Reason of no surgery, Radiation sequence with surgery	Age, Number of primaries
Palaniappan and Hong [44]	Race, Marital status, Primary site code, Histologic type, Behavior code, Grade, Extension of tumor, Lymph node involvement, Radiation, Stage of cancer,	Age, Tumor size, Number of positive nodes, Number of Nodes, Number of primaries
Khan et al. [6]	Race, Marital status, Primary site, Histologic Type ICD, Behavior Code, Grade, Extension of tumor, Node Involvement, Radiation, Stage, Site-specific Surgery	Age at diagnosis, Tumor size, Number of positive nodes, Number of Nodes, Number of primaries
Liu et al. [11]	Race, Marital status, Primary site code, Histology, Grade, Extension of disease, Lymph node involvement, Radiation, Stage of cancer, Site-specific surgery code, first malignant	Age, Tumor size, Number of positive nodes, Number of Nodes, Number of primaries
Ali et al. [45]	Race, Marital status, Primary site, Histologic Type, Behavior Code, Grade, Extension of tumor, Lymph Node Involvement, Radiation, Stage, Site-specific Surgery	Age at diagnosis, Tumor size, Number of positive nodes, Number of Nodes, Number of primaries

Table S2 Summary of predictor variables for survivability in the literature

Categorical variables		Number of distinct values			
**Variable ID in the study**	**Label**				
re_v4	Race	27			
re_v3	Marital status	5			
re_v14	Primary site code	9			
re_v19	Behavior Code ICD-O-3	2			
re_v20	Grade	4			
re_v24	Extension of disease	32			
re_v26	Lymph node involvement	9			
re_v62	Reason of no surgery	5			
re_v63	Radiation	9			
re_v65	Radiation sequence with surgery	7			
re_v96	Histology	19			
re_sss	Site-specific surgery code	9			
re_v102	Stage of cancer	4			
re_v104	SEER modified AJCC stage 3rd	9			
re_v108	First malignant	2			
**Numerical variables**		**Mean**	**S.D.**	**Min.**	**Max.**
v8	Age	58.91	13.28	18	106
v23	Tumor size	20.70	16.24	0	200
v27	Num of positive nodes	1.44	3.69	0	79
v28	Num of nodes	13.11	7.66	1	90
v107	Num of primaries	1.25	0.52	1	9
